# Geometric Analysis of Alloreactive HLA ***α***-Helices

**DOI:** 10.1155/2014/943186

**Published:** 2014-06-17

**Authors:** Reiner Ribarics, Rudolf Karch, Nevena Ilieva, Wolfgang Schreiner

**Affiliations:** ^1^Section of Biosimulation and Bioinformatics, Center for Medical Statistics, Informatics and Intelligent Systems (CeMSIIS), Medical University of Vienna, Spitalgasse 23, 1090 Vienna, Austria; ^2^Institute for Nuclear Research and Nuclear Energy (INRNE), Bulgarian Academy of Sciences 72, Tzarigradsko Chaussee, Sofia 1784, Bulgaria

## Abstract

Molecular dynamics (MD) is a valuable tool for the investigation of functional elements in biomolecules, providing information on dynamic properties and processes. Previous work by our group has characterized static geometric properties of the two MHC *α*-helices comprising the peptide binding region recognized by T cells. We build upon this work and used several spline models to approximate the overall shape of MHC *α*-helices. We applied this technique to a series of MD simulations of alloreactive MHC molecules that allowed us to capture the dynamics of MHC *α*-helices' steric configurations. Here, we discuss the variability of spline models underlying the geometric analysis with varying polynomial degrees of the splines.

## 1. Introduction

Major histocompatibility complexes (MHC) play a key role in immune reactions. The function of this class of highly polymorphic proteins is to bind peptide fragments (p) derived from pathogens or tumour antigens and display them on the cell surface for recognition by appropriate T cells. T cells can detect these peptide fragments from pathogens or cancer cells by T cell receptor (TCR) molecules on their cell surface, but only if these peptides are presented in complex with MHC molecules (pMHC). As a consequence of the TCR/pMHC interaction, pathogen-infected cells or cancer cells can be detected and eliminated by the immune system.

**Figure 1:**
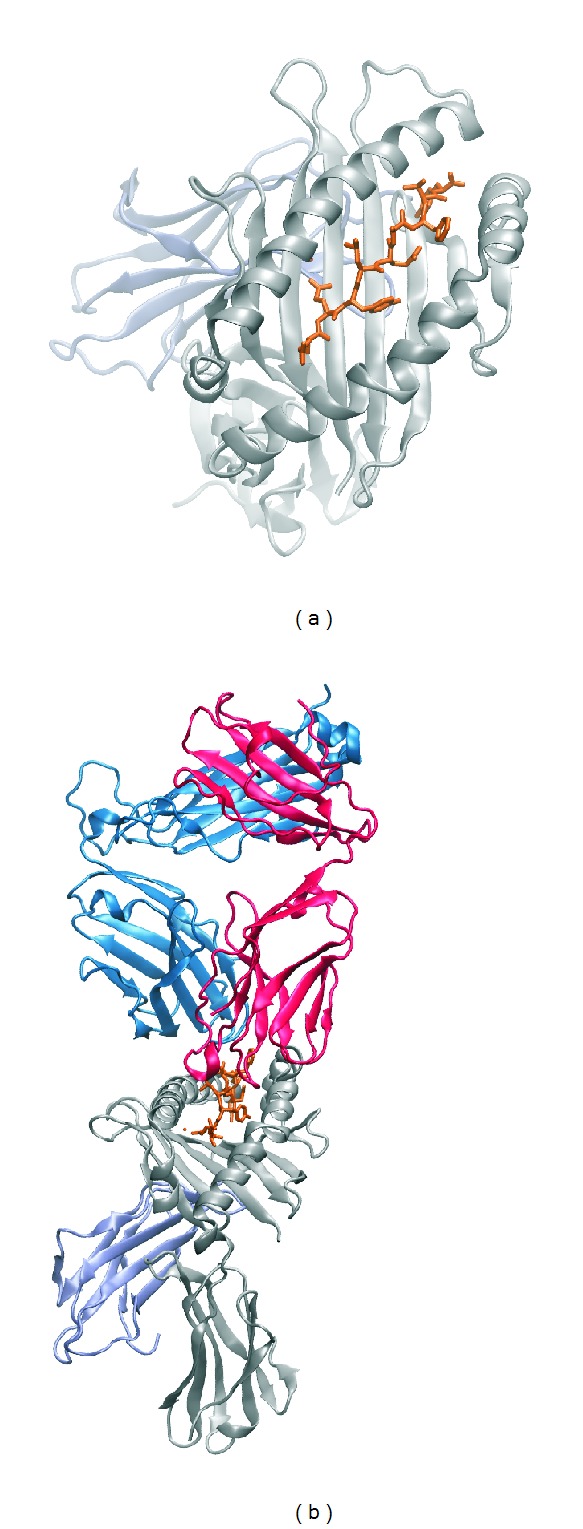
*Molecular structure of MHC class I*. Three-dimensional representation of secondary structural elements of. (a) HLA-B^∗^44:05 (grey), ABCD3 peptide (orange), and *β*_2_-microglobulin (ice blue). (b) HLA-B^∗^44:05 (grey), ABCD3 peptide (orange), *β*_2_-microglobulin (ice blue), and LC13 T cell receptor (dark blue and red). PDB ID: 3KPS.

The peptide binding region of class I MHC molecules comprises two *α*-helices and a *β*-sheet as a floor. The *α*-helices are orientated in an antiparallel manner to form a binding pocket (see Figure 1).

The TCR is a heterodimer comprising one *α*- and one *β*-chain. Each chain has a constant and a variable domain. The constant domain is facing the cell membrane, while the variable domain is facing the extracellular space ready for interaction with MHC molecules. The area of interaction between these two proteins comprises the two MHC *α*-helices and the three hypervariable complementarity determining regions (CDR) 1, 2, and 3 of the TCR. The peptide in the MHC binding groove mainly interacts with the TCR via CDR 3.

TCR and MHC molecules show wide diversity, therefore sophisticated selection mechanisms exist to prevent autoreactivity that could lead to autoimmunity. During development, T cells are restricted to only recognize host MHC [1, 2]. In other words, T cells only recognize cognate antigen presented by one of the MHC molecules that are present in the host in which they have developed. These T cells, however, may directly react with MHC molecules that are not present in the host (allogeneic reaction). T cells form the basis of allograft rejection, where the host immune system recognizes the transplant as an intruder due to allogeneic MHC molecules.

With molecular dynamics (MD) simulations it is possible to simulate the physical movements of atoms and molecules by solving Newton's equations of motion. The simulations can be used to investigate functional molecular elements and dynamic molecular processes, for example, signal transduction [3–7]. We analyse molecular dynamics simulations of three closely related MHC molecules of the HLA class B44: HLA-B^∗^44:02, HLA-B^∗^44:03, and HLA-B^∗^44:05. Each MHC molecule harbours the self-antigen ABCD3 in its antigen binding groove and is ligated to the LC13 T cell receptor. Of note, the LC13 TCR alloreacts with HLA-B^∗^44:02 and HLA-B^∗^44:05, but not with HLA-B^∗^44:03. This fact is also reflected in the binding affinities of the respective TCR/pMHC complexes: LC13 binds HLA-B^∗^44:02 and HLA-B^∗^44:05 with high affinity, whereas binding HLA-B^∗^44:03 is very weak [8].

Previous work by our group characterized the geometric properties of MHC *α*-helices of a plethora of static crystal structures [9–11] found in the protein database (PDB, [12]). The aim of the present work is to describe the dynamics of the MHC *α*-helices in the above-mentioned set of allogeneic HLA-types using spline representation. Spline representation allows to mathematically represent the overall shape of the MHC *α*-helices and capture their geometric properties over the simulation time. The mathematical description of structural elements of macromolecules has been used before, for example, for visualization [13], for calculation of differential geometric parameters of helix bundles [14], and for monitoring of structural changes of Leucine-Rich Repeat (LRR) proteins [15].

We focus on the analysis of geometric mathematical quantities of the MHC *α*-helices that allow us to characterize their shape and geometry, that is, interhelical distance and area of the ruled surface spanned by the MHC *α*-helices. We also discuss the variability of spline models with polynomial degrees *m* = 2,3, 4 and *K* = 0 interior knots.

## 2. Methods

### 2.1. Construction of Complexes for Molecular Dynamics Simulation

Conformational transitions occur on a variety of time scales ranging from nanoseconds to seconds [13]. This work represents a proof of concept study for geometrical representation of MHC *α*-helices, hence, molecular dynamics simulations were performed for 250 ns. A simulation time of 250 ns is a feasible choice for such large systems of about 400.000 atoms (proteins and solvent) as shown in Table 1.

HLA-B^∗^44:05 plus ABCD3 peptide (EEYLQAFTY) ligated to the LC13 TCR have been successfully crystallized by Macdonald et al. [8]. The structure is accessible on http://www.pdb.org/ (PDB ID 3KPS). Unfortunately, the structures of HLA-B^∗^44:02 and HLA-B^∗^44:03 plus ABCD3 peptide and LC13 TCR have not been resolved. Therefore, we used the technique of homology modelling to create the missing structures.

**Figure 2:**
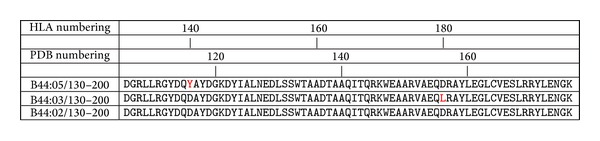
Alignment of amino acid sequences of HLA-B^∗^44:02, HLA-B^∗^44:03, and HLA-B^∗^44:05 (downloaded from IMGT/HLA database [14]). HLA-B^∗^44:05 was used as a template, because a three-dimensional structure of this MHC in complex with ABCD3 peptide and LC13 TCR was available. Sequence alignment was done with CLC bio's sequence viewer. Note that PDB sequence numbering and IMGT/HLA database numbering differ.

For generation of LC13/ABCD3/HLA-B^∗^44:03 (complex of TCR/pMHC) we used PDB structure 3KPS as a template. As mentioned above, this structure file includes LC13/ABCD3/HLA-B^∗^44:05. In order to change the HLA type from B^∗^44:05 to B^∗^44:03 we introduced Y116D and D156L mutations into the MHC molecule (amino acid positions specified by PDB numbering; see Figure 2). To modify the crystal structure and substitute amino acids, that is, in silico mutagenesis, we used the Swiss PDB Viewer. This program allows users to change amino acid side-chains and automatically browses a rotamer library to select that rotamer minimising a scoring function. Rotamers are defined as low-energy side-chain conformations. However, the rotamer energy optimisation by the scoring function only works locally and can, in certain circumstances, result in clashes, that is, atoms come into close contact so that the repulsion term of the Lennard-Jones potential predominates. Proper energy minimisation is routinely performed in the subsequent molecular dynamics simulation protocol (we used a steepest-descent method).

For the generation of LC13/ABCD3/HLA-B^∗^44:02 we again used PDB structure 3KPS as a template. In order to change the HLA allele from B^∗^44:05 to B^∗^44:02, we introduced the Y116D mutation into the MHC molecule (see Figure 2 for sequence alignment) using in silico mutagenesis.

### 2.2. Molecular Dynamics Simulation Protocol

MD simulation of TCR/pMHC systems (B4402, B4403, and B4405) was performed using GROMACS 4.0.7 [15] according to the following protocol.

First, we immersed the TCR/pMHC complex in a SPC [16] artificial water bath (cubic box) allowing for a minimum distance of 2 nm between complex and box boundaries. Second, we added sodium and chloride ions to a concentration of 0.15 mol/L and at the same time neutralized the net charge of the system. Third, we minimized the energy of the solvated system using a steepest descent method. Next, we warmed up the system to 310 K during a 100 ps position-restraints MD simulation. Finally, we carried out MD production runs with LINCS constraint algorithm acting on all bonds and using the GROMOS96 53a6 force field [17]. Hydrogen motions were removed allowing for an integration step of 5 fs. Coordinates were written to disk every 50 ps of simulation time. Coulomb interactions were computed using Particle Mesh Ewald (PME) with a maximum grid spacing of 0.12 nm and interpolation order 4. Van der Waals and Coulomb interactions were computed with a cut-off at 1.4 nm. Velocity rescale temperature coupling was set to 310 K and Berendsen isotropic pressure coupling was set to 1 bar. All other parameters were set in accordance with Omasits et al. [18].

### 2.3. Spline Representation of MHC *α*-Helices

The* MH* ^*2*^ *C* software package introduced by Hischenhuber et al. [11] provides a general approach to model *α*-helices of any macromolecule containing this secondary structural element. Molecular dynamics simulations of TCR/pMHC complexes yield a series of time evolving molecular conformational structures. The resulting structures were subjected to analysis by* MH* ^*2*^ *C*. As mentioned in the introduction, MHC molecules comprise two *α*-helices, hereafter named G-ALPHA1 helix and G-ALPHA2 helix. In order to mathematically describe and quantify the helical movements, spline curves $\overset{⃑}{c}(z)$ are fitted to the *α*-helices where *z* is the curve parameter. To do that, we extracted the *C*_*α*_ atom coordinates of the *α*-helices' amino acids, which are in accordance with the classification of *α*-helices of visual molecular dynamics (VMD [19] implementing the STRIDE [20] and DSSP [21] algorithms). In* MH* ^*2*^ *C* each helix is first subjected to a principal component analysis (PCA), yielding three principal components PC1, PC2, and PC3. These are used as a local coordinate system (“reference frame” of the respective helix) for least-square approximation of the *C*_*α*_ atom coordinates by two spline functions: *f*_2_ in the plane PC1-PC2 and *f*_3_ in the plane PC1-PC3, as done in our previous investigation [11]:

(1) $$\begin{array}{c} \overset{⃑}{c}\left(z\right)=c=\left(\begin{array}{c} z\\ f_{2}\left(z\right)\\ f_{3}\left(z\right) \end{array}\right). \end{array}$$

Here, we study splines *f*_2_, *f*_3_ with *K* = 0 interior knots, that is, we consider only one single spline segment comprising polynomials *P*[*m*] = {*p* : *R* → *R*∣*p*(*x*) = ∑_*i*=0_^*m*^ *a*_*i*_ *x* ^*i*^;   *a*_*i*_ ∈ *R*, *i* = 0,…, *m*} of degrees *m* = 2,3, 4. We refrained from using interior knots and set = 0, yielding a total of three models.

### 2.4. Global Geometric Quantities

The* MH* ^*2*^ *C* software package was used to extract global shape characteristics of the MHC molecule, which are less affected by short-term fluctuations in time, as compared to single helix parameters. Each helix is represented by a spline, and the interhelical area is represented by a surface defined by “rulings” (i.e., straight lines) spanned between corresponding points (opposite to each other) on these splines [9]. We use *M* rulings (1200 ≤ *M* ≤ 1500) parameterized by a common parameter *u*.

#### 2.4.1. Interhelical Distance and Area of Interhelical Surface

Rulings between splines of the two *α*-helices **c**_1_ and **c**_2_ (each assigned identical polynomial degrees *m*) lend themselves for a straightforward triangulation of the ruled surface [22]. From distances between splines

(2) $$\begin{array}{c} d\left(u_{i}\right)=||c_{2}\left(u_{i}\right)-c_{1}\left(u_{i}\right)||\;1\leq i\leq M \end{array}$$

and distances between rulings, the total intrahelical area, *A*, is computed as outlined in [9]. Likewise, median, quartiles, and extreme values (boxplots) of *d*(*u*_*i*_) over time are calculated for each *i*; see Section 3.1. These graphs provide a rough estimate of changes in width of the intrahelical gap (i.e., the binding cleft) both as a function of helical position and of time.

## 3. Results

**Figure 3:**
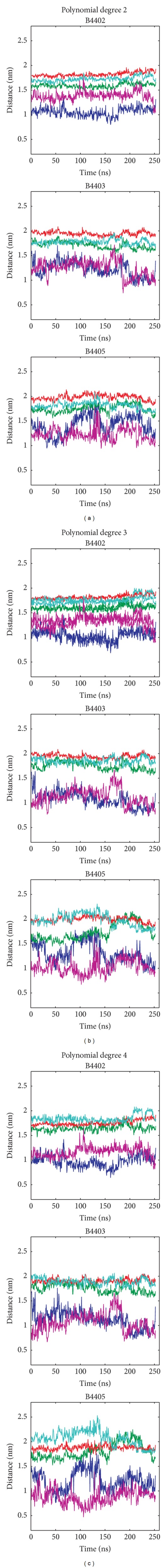
*Interhelical distances between MHC α* *-helices*. Each spline is discretised at about 1500 coordinate points. Interhelical distances between spline positions 1, 369, 737, 1105, and 1471 (blue, green, red, cyan, and magenta, resp.,) were evaluated along a 250 ns MD trajectory for three different molecular systems (B4402, B4403, and B4405) and three different polynomial degrees (*m* = 2, (a); *m* = 3, (b); and *m* = 4, (c)).

Three spline models of different polynomial degrees were applied to fit the MHC *α*-helices of three different molecular systems yielding a total of nine time series per global quantity (see Figures 3 and 5). From the graphs we get an impression of how interhelical distances and the total intrahelical area, *A*, are affected by different polynomial degrees of the spline functions *f*_2_ and *f*_3_.

### 3.1. Interhelical Distances

**Figure 4:**
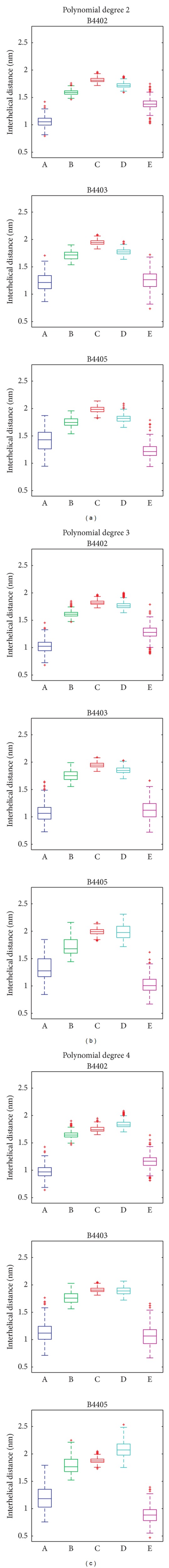
*Boxplots of interhelical distances between MHC α* *-helices.* Each spline is discretised at 1500 coordinate points. Boxplots of interhelical distances between spline positions 1, 369, 737, 1105, and 1471 (blue, green, red, cyan, and magenta, resp., A, B, C, D, and E) are shown along a 250 ns MD trajectory for three different molecular systems (B4402, B4403, and B4405) and three different polynomial degrees (*m* = 2, (a); *m* = 3, (b); and *m* = 4, (c)).

Interhelical distances were measured between five selected points on the splines fitted to G-ALPHA1 helix and G-ALPHA2 helix for polynomial degrees *m* = 2,3, 4. Each spline was discretised at 1500 discrete coordinate positions from which 1, 369, 737, 1105, and 1471 were selected to describe one aspect of the global shape of the MHC's helical interface (others could include, for example, spline curvature and torsion). Positions 1 and 1471 represent the spline ends or flanking points. Positions 269, 737, and 1105 represent three points of the central part of the splines. On the one hand, in all simulations, the flanking points' distances show the largest fluctuations, but on average are smaller than at the other positions. This reflects the helical bending as seen in Figure 1, panel A. On the other hand, the central parts of the splines show rather little motility for complexes B4402 and B4403 across all models, as seen in Figure 3. For B4405, the central points at positions 369 and 737 show larger fluctuations for models with polynomial degrees 3 and 4 than for the model with degree 2; see Figure 4.

### 3.2. Area of Ruled Surface between MHC *α*-Helices

**Figure 5:**
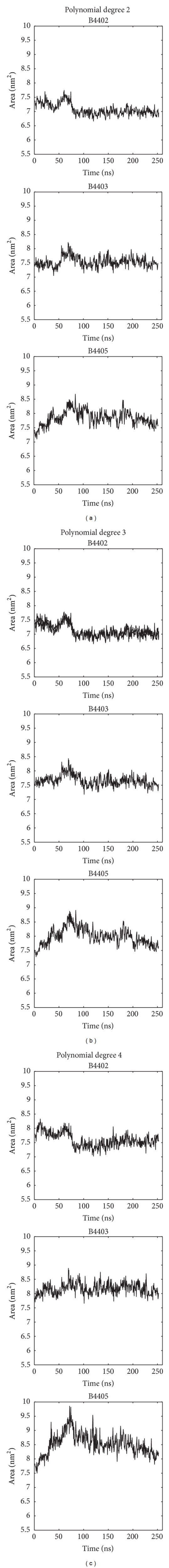
*Area of ruled surface between MHC α* *-helices*. From distances between splines and distances between rulings, the total intrahelical area, *A*, is computed along a 250 ns MD trajectory for three different molecular systems (B4402, B4403, and B4405), and three different polynomial degrees (*m* = 2, (a); *m* = 3, (b); and *m* = 4, (c)).

The area of the ruled surface between MHC *α*-helices, *A*, was measured between splines fitted to G-ALPHA1 helix and G-ALPHA2 helix for polynomial degrees *m* = 2,3, 4 for three different MD simulations. The time course of area *A* is similar for polynomial degrees 2 and 3 (see Figure 5). However, polynomial degree 4 shows an increase of the time averaged area, *A*: 6.7% for B4402, 7.5% for B4403, and 7.6% for B4405.

## 4. Discussion

The restriction of T cells to host MHCs is the key mechanism preventing autoimmunity. However, a different shape of an MHC might trigger an immune reaction, even when loaded with self-peptide. Hence, it is of focal interest to spot such changes in MHC geometry, for which we have proposed our geometrical modelling [9]. In the current work, we present a pilot study on geometrical quantities related to the interhelical area that directly interacts with the T cell receptor that could induce signal transduction.

The new approach in this work is to consider not only single static configurations of MHCs, but to include dynamics. Each specific MHC changes its shape continuously, due to thermal movement. However, these differences in shape do not trigger restricted TCRs. There need to be differences shining through all these thermal movements and becoming relevant in the long MD simulation run.

In our previous work model flexibility was investigated for splines fitted to single helices, which, by their nature, exhibit rather large fluctuations due to motions of small groups of atoms. Here, we investigate model flexibility related to geometric quantities (i.e., interhelical distances and total area between helices) which by their nature resemble more global features of a molecule and should be less affected by stochastic motions of small groups of atoms. Increasing flexibility seems to add short term fluctuations. The question is if these correspond to actual movements of the helices, which are relevant for interpretation, or if they are just artefacts of overfitted models. The impact of increased model flexibility was investigated for rather insensitive quantities such as interhelical distances and total area between MHC *α*-helices.

Our goal is to detect differences in the dynamics of the TCR/pMHC complexes originating from different MHC molecules (HLA-alleles B^∗^44:02, B^∗^44:03, and B^∗^44:05). In order to achieve that we need to select a reliable model that fits the data accurately and reflects helical motions. As described in our previous work, a reliable model can be selected by the Akaike information criterion [11]. However, automation of model selection holds the risk that different spline models will be selected for different MD simulations. How can we know if variability between different MD simulations is really associated with the different natures of the TCR/pMHC complexes or if the variability results from different spline models? In order to prevent the helix representation from adapting configurations that would not make sense from a physicochemical point of view, one should use polynomials of low degrees. In our case, high polynomial degrees would result in fitting the helix turns that would affect our interpretation of global parameters as well as differential geometric parameters.

For the area, *A*, of the ruled surface between MHC *α*-helices, the models with polynomial degrees 2 and 3 yield rather similar mean values. However, the model with degree 4 shows a roughly 7% increase in *A*, when compared to lower polynomial degrees. Since *A* is sensitive to model selection, one has to be careful when comparing mean values of *A* across different simulations. However, the shape of the time series of *A* is similar across all models, indicating that *A* is a quantity rather insensitive to small fluctuations during an MD simulation. For *A* we suggest to use the same model when one wants to compare *A* between different simulations of similar molecular systems.

For the other quantity described in this work, the interhelical distances, the situation is different; for example, the interhelical distances are comparable between all models for B4403 (Figure 4, second row). However, the same distances show large variations and fluctuations between all models for HLA-B^∗^44:05 (Figure 4, third row). Therefore, we suggest performing a careful analysis before comparing values across different MD simulations. In future studies one could further evaluate the helical dynamics and see if the range of helical structures is well preserved or rather transient in nature over time.

## Figures and Tables

**Table 1:** Molecular systems simulated.

Molecular system	Simulation length
LC13 TCR/ABCD3/HLA-B∗44:02 (B4402)	250 ns
LC13 TCR/ABCD3/HLA-B∗44:03 (B4403)	250 ns
LC13 TCR/ABCD3/HLA-B∗44:05 (B4405)	250 ns
